# Complexity of the Inoculum Determines the Rate of Reversion of SIV Gag CD8 T Cell Mutant Virus and Outcome of Infection

**DOI:** 10.1371/journal.ppat.1000378

**Published:** 2009-04-10

**Authors:** Liyen Loh, Jeanette C. Reece, Caroline S. Fernandez, Sheilajen Alcantara, Robert Center, Jane Howard, Damian F. J. Purcell, Mehala Balamurali, Janka Petravic, Miles P. Davenport, Stephen J. Kent

**Affiliations:** 1 Department of Microbiology and Immunology, University of Melbourne, Melbourne, Victoria, Australia; 2 Center for Vascular Research, University of New South Wales, Sydney, Australia; University of Pennsylvania School of Medicine, United States of America

## Abstract

Escape mutant (EM) virus that evades CD8+ T cell recognition is frequently observed following infection with HIV-1 or SIV. This EM virus is often less replicatively “fit” compared to wild-type (WT) virus, as demonstrated by reversion to WT upon transmission of HIV to a naïve host and the association of EM virus with lower viral load *in vivo* in HIV-1 infection. The rate and timing of reversion is, however, highly variable. We quantified reversion to WT of a series of SIV and SHIV viruses containing minor amounts of WT virus in pigtail macaques using a sensitive PCR assay. Infection with mixes of EM and WT virus containing ≥10% WT virus results in immediate and rapid outgrowth of WT virus at SIV Gag CD8 T cell epitopes within 7 days of infection of pigtail macaques with SHIV or SIV. In contrast, infection with biologically passaged SHIV_mn229_ viruses with much smaller proportions of WT sequence, or a molecular clone of pure EM SIV_mac239_, demonstrated a delayed or slow pattern of reversion. WT virus was not detectable until ≥8 days after inoculation and took ≥8 weeks to become the dominant quasispecies. A delayed pattern of reversion was associated with significantly lower viral loads. The diversity of the infecting inoculum determines the timing of reversion to WT virus, which in turn predicts the outcome of infection. The delay in reversion of fitness-reducing CD8 T cell escape mutations in some scenarios suggests opportunities to reduce the pathogenicity of HIV during very early infection.

## Author Summary

Understanding how to contain HIV replication by the immune system is a key goal of vaccine strategies. HIV frequently mutates to avoid immune recognition, but this may come at a “fitness cost”, weakening the virus. When HIV is transmitted to a new host, the mutations often revert back to wild-type, allowing the virus to regain a fitter state. We found that when multiple HIV-like viruses are transmitted to monkeys, containing both mutant and wild-type, reversion to wild-type is very rapid and the fitter virus results in higher viral levels. In contrast, when only escape mutant virus initiates the infection, reversion to wild-type is delayed to later during early infection, and lower levels of virus result. Our results suggest that the composition of the infecting virus plays a role in determining the outcome of HIV infections. Strategies to maintain weakened virus strains during the early HIV infection may help the host control virus replication.

## Introduction

The high mutation rate of HIV coupled with the strong immune selection pressure exerted by CD8+ T lymphocytes leads to the frequent selection of ‘escape mutant’ (EM) virus that contains mutations within CD8+ T cell epitopes that abrogates their recognition and killing by T cells. Although these mutations confer an advantage on EM virus, they also incur a ‘fitness cost’, as EM virus often has lower replicative capacity than the original wild-type (WT) virus, particularly when mutations occur in conserved regions such as Gag [1],[2]. This reduced replicative capacity of EM virus leads to reversion from EM to WT virus in HIV-infected humans and SIV-infected macaques following transmission to naïve hosts that lack the appropriate MHC alleles to recognise the epitope concerned [3]–[6]. There is strong evidence from a recent study of over 100 human HIV-1 transmissions that lack of reversion at multiple key Gag CD8 T cell epitopes is associated with lower viral loads [7]. Thus, the fitness costs of escape mutations contribute to partial viral control even when the epitope is not recognised [8].

The rate of reversion from EM to WT *in vivo* is however highly variable. Factors that impact the rate of immune escape and reversion include the particular CD8 T cell epitope, target cell availability, prior vaccination, the presence of compensatory mutations, the MHC of the donor and recipient, and timing of appearance of the mutation [7], [9]–[13]. Both escape and reversion have been observed to occur extremely rapidly in experimental infection of macaques, but generally much more slowly in natural HIV-1 infection. It has been suggested that the slow dynamics of escape and reversion in HIV-1 implies lower immune pressure and lower fitness costs of escape in HIV-1 [14]. However, this analysis ignores potential differences in the size and diversity of the initial inoculum in natural HIV and experimental SIV infection. Earlier studies of HIV-1 infection in humans strongly suggested many, but not all, transmission events are initiated with single virus quasispecies [15]–[24]. Recent data using single genome amplification strategies of over 100 subjects with acute HIV-1 infection suggests that most (76%) subjects with HIV-1 infection acquire just a single viral strain, while the rest (24%), acquire 2 or more variants [25]. The ability of minor WT variants to outgrow a dominant EM variant is difficult to evaluate in humans where the infecting HIV-1 isolate(s) and timing of infection are not precisely known and availability of samples prior to the peak of acute infection is limited. Macaques can be infected with known proportions of EM and WT SIV or SHIV viruses to accurately study *in vivo* competition between EM and WT variants under different conditions.

A considerable technical hurdle in evaluating the evolution of immune escape variants is sensitively and specifically quantifying minor variants. Cloning and sequencing is poorly quantitative for variants of <10% of the total unless prohibitively large numbers of clones are analyzed. We recently developed quantitative real-time PCR assays (qRT-PCRs) to study escape mutations that emerge at 2 different SIV Gag CD8 T cell epitopes (KP9 and AF9) in pigtail macaques [10],[12]. These assays are sensitive to variants comprising ≤0.01% of total virus, levels that would be almost impossible to quantify with standard cloning and sequencing techniques. This advance now allows us to quantify the evolution of both the WT and EM variants following inoculation with different mixes of WT and EM virus. Specifically, we examined how the ratio of EM∶WT virus in the infecting inoculum affected the dynamics of reversion *in vivo* and the impact of this on viral load in acute and chronic infection.

## Methods

### Animals

Experiments on outbred pigtail macaques (*Macaca nemestrina*) were approved by the University of Melbourne and CSIRO livestock industries Animal Ethics Committees. Pigtail macaques were typed by reference strand-mediated conformational analysis for the MHC-I alleles Mane-A*10 and Mane-A*17 allele which present SIV Gag_164–172_ KP9 epitope and SIV Gag_371–379_ AF9 epitope respectively [26],[27].

### SHIV_mn229_ infection

Reversion of KP9 EM SHIV_mn229_ viruses (K165R) from 3 different viral inocula derived from pigtail macaques expressing the restricting Mane-A*10 allele were assessed following passage of each virus to 2–4 naïve Mane-A*10 negative pigtail macaques. We studied reversion of the original SHIV_mn229_ stock in 4 animals as previously described [10]. This original X4-tropic SHIV_mn229_ stock was derived following passage of SHIV_HXB2_ in Mane-A*10 positive pigtail macaques and is 89% EM and 11% WT at the K165 position within the Gag KP9 epitope [5],[10]. The typical escape mutation at the KP9 epitope is a lysine to arginine change at amino acid 165 of Gag (K165R).

Reversion of the K165R mutation back to WT was also studied by transmission of further passages of the SHIV_mn229_, directly from the serum / cells of two infected animals that showed escape at the KP9 epitope. This passaged virus contained a much lower level of WT virus than the original SHIV_mn229_ stock, and was inoculated into 4 additional Mane-A*10 negative macaques. Two macaques (6274 and 6366) received 1 ml of plasma and 3×10^6^ peripheral blood mononuclear cells (PBMC) from the donor Mane-A*10+ animal 4296 8 weeks after SHIV_mn229_ infection. Two additional macaques received plasma and serum samples from Mane-A*10 positive animal 6279 11 weeks after SHIV_mn229_ infection. Animals 4296 and 6279 were previously described as part of a DNA and Fowlpoxvirus vaccine studies in macaques [28],[29]. The DNA and Fowlpoxvirus vaccines expressed WT SIV Gag, inducing KP9 specific CD8 T cells which force further escape at KP9 following SHIV_mn229_ challenge [9]. Plasma samples transferred at these time points contained 4.0% and 0.34% WT virus respectively by qRT-PCR as described below.

To assess the pathogenicity of the EM viruses, we compared viral loads of the passaged low WT SHIV_mn229_ with 21 unvaccinated control pigtail macaques previously infected with the original SHIV_mn229_ stock (89∶11 EM∶WT) previously used across several vaccine studies [28]–[30].

### SHIV_SF162P3_ AF9 mutant virus

Reversion of AF9 was studied as previously described [31] in two naive Mane-A*17 negative macaques infected with SHIV_SF162P3_ passaged *in vivo* in a Mane-A*17+ pigtail macaque. This passaged SHIV acquired a 6-nucleotide deletion within the AF9 epitope [31]. The transmitted plasma contained approximately 50% WT virus and 50% EM virus at AF9 when measured by qRT-PCR as described below [12].

### K165R SIV_mac239_ molecular clone

To examine the effect of infection with pure pathogenic R5-tropic KP9 EM virus, we used PCR mutagenesis techniques to mutate the translated amino acid from lysine to arginine at Gag position 165 within our previously described SIV_mac239_ proviral plasmid [32]. Briefly, a specific mutation was introduced into the 9.9 kb 5′ plasmid [32],[33] using the QuikChange™ II site-directed mutagenesis kit according to the manufacturer's protocol (Stratagene, La Jolla, CA). using the primers 5′ -GGTAAAATTGATAGAGGAAAAGAGATTTGGAGCAGAAGTAGTGCC-3′ and 5′- GGCACTACTTCTGCTCCAAATCTCTTTTCCTCTATCAATTTTACC-3′. The mutated 5′ vector and 3′ vector of SIV_mac239_ [32] were joined together by digesting with Sph I, followed by ligation and transformation using TOP10 cells. The authenticity of the resulting constructs were confirmed by double-stranded DNA sequencing. We confirmed the K165R EM SIV_mac239_ DNA was infectious *in vitro* by transfecting plasmids into HeLa cells and testing the supernatants for their ability to infect CEMX174 *in vitro* (not shown).

To initiate infections *in vivo*, we injected animals with 150 µg of the K165R SIV_mac239_ proviral DNA intramuscularly, a technique we previously used to initiate infections with both WT and nef/LTR-deleted SIV_mac239_ strains [32]. To model the effect of dual WT and EM infection (similar to the biological SHIV_mn229_ stock containing 11% WT virus at KP9), we also inoculated 2 animals IM with 150 ug of a 90∶10 mix of K165R EM and WT SIV_mac239_ proviral plasmids.

### Quantitative real-time PCR

To quantify virus levels of WT or EM quasispecies at the KP9 and AF9 epitopes we employed recently published novel real-time PCR assays [10],[12]. The assays use a forward primer specific for either wild-type sequence or specific for the nucleotide mutation encoding the dominant K165R KP9 escape mutant or 6-nucleotide deletion AF9 escape mutant. In short, for each time-point after infection 10 µl of RNA extracted from EDTA-anticoagulated plasma was subjected to reverse-transcription to create cDNA that was then amplified by qRT-PCR using either WT or EM primers specific for the appropriate SIV Gag epitope. A reverse primer and 5′ 6FAM labelled Minor Groove Binding (MGB)-DNA probe were also added for quantification against the appropriate SIV Gag epitope RNA standards using the ABI Prism 7700 sequence detection system PCR thermal cycler or an Eppendorf Realplex^4^ cycler. Analysis was performed using SDS applications version 1.9 (ABI) or Eppendorf Realplex^4^ software. Baselines were set 2 cycles earlier than real reported fluorescence and threshold value was determined by setting threshold bar within the linear data phase. Samples amplifying after 40 cycles were regarded as negative, and corresponds to <1.5-Log_10_ SHIV/SIV RNA copies/ml of plasma (threshold value of quantification).

### Rates of reversion

In order to compare the dynamics of reversion in different animals we analysed reversion rates as previously described [5]. We defined reversion rate as the absolute difference in growth rates between the wild type (W) and mutant (M) in a given time interval. Briefly, if *f_W_*(*t*) is the fraction of wild type and *f_M_*(*t*) is the fraction of escape mutant clones at time *t*, we calculate the time-dependent reversion rate *R* from the proportions of clones of each type at the end points *t_s_* and *t_e_* of the time interval (*t_s_*, *t_e_*),
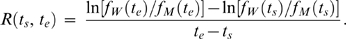
(1)


### The model of reversion

In order to explain the experimental results, we used a standard model of viral dynamics applied to reversion [11]. In this model, wild type (*W*) and escape mutant (*M*) virus with different, but constant in time, replicative capacities, infect the same pool of target cells *T* (in the case of SHIV_mn229_ these are all CD4+ T cells), generating infected cells *I_W_* and *I_M_* respectively. This model is described by the set of equations:
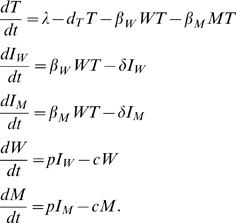
(2)In the system of Equation 2, target cells *T* are replaced from an external source at the rate *λ* and in the absence of infection are lost at the rate *d_T_*. The difference in replicative capacities of WT and EM is the result of different rates of infection of target cells (*β_W_* and *β_M_* respectively, where *β_W_*>*β_M_*). Cells infected by both strains die at the same rate *δ* (since there is no immune response specific to one strain). Both strains are produced from infected cells at the same rate *p* and are cleared at the same rate *c*.

This model predicts the reversion rate in the time interval between *t_s_* and *t_e_*,
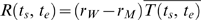
(3)where *r_W_* = *β_W_p/c* and *r_M_* = *β_M_p/c* are the replicative capacities of WT and EM respectively, and 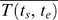 is the average number of target cells in the interval (*t_s_*, *t_e_*). The model gives the reversion rate directly proportional to target cell number.

## Results

**Figure 1:**
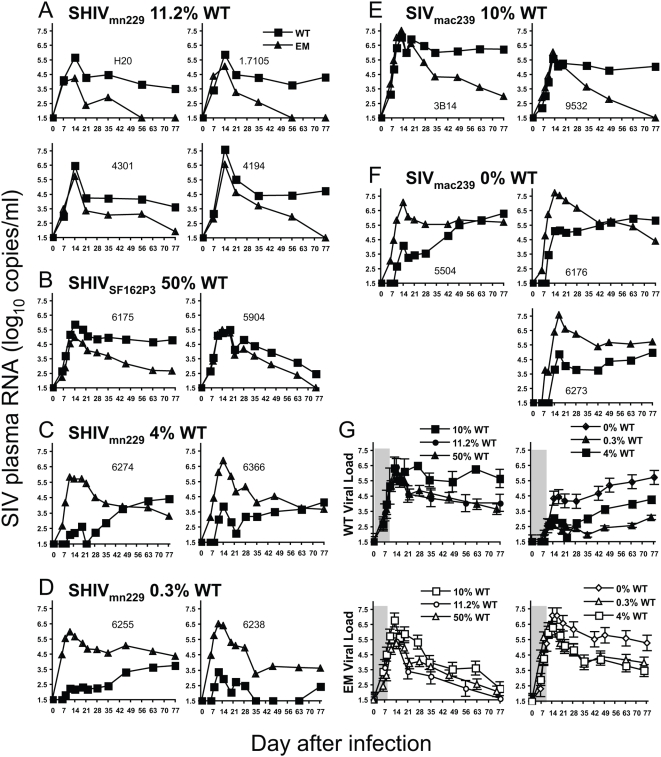
Reversion to WT for different viruses and percentages of WT in the inocula. (A–F) Shows WT (squares) and EM (triangles) plasma viral loads over time by qRT-PCR from individual pigtail macaques inoculated with different viruses over 11 weeks. (A) SHIV_mn229_ stock with 11.2% WT virus at KP9 CD8 T cell epitope (four animals) (B) *In vivo* passage of SHIV_SF162P3_ with 50% WT virus at AF9 CD8 T cell epitope (2 animals). (C) *In vivo* passage of SHIV_mn229_ with 4.0% WT virus at KP9 (2 animals). (D) *In vivo* passage of SHIV_mn229_ with 0.34% WT virus at KP9 (two animals). (E) Mix of SIV_mac239_ molecular clones containing 10% WT virus and 90% K165R EM virus. (F) Pure SIV_mac239_ molecular clone of 100% K165R EM virus (0% WT, 3 animals). (G) Mean (±SEM) of WT (upper panels) or EM (lower panels) viral loads of groups of animals given the same virus. Animals administered mixes of EM and WT virus with ≥10% WT have similar WT and EM viral loads and are grouped together (left panels) in comparison to animals administered viruses with <10% WT content (right panels). The first 10 days are shaded to indicate the differences in WT virus expansion between the two types of viruses.

A fitness cost is usually incurred by CD8 T cell escape mutations; this is most clearly demonstrated when EM virus reverts to the fitter WT upon transmission to MHC mismatched hosts [3]–[5],[34]. Rapid reversion of EM KP9 virus occurred in Mane-A*10 negative macaques infected with our SHIV_mn229_ viral stock (Figure 1A). This biological isolate is 11.2% WT by qRT-PCR (9.1% by cloning and sequencing 44 clones) since it was derived from an infectious SHIV_HXB2_ clone originally passaged in Mane-A*10+ pigtail macaques [5]. In our first analysis, we compared reversion to WT of our stock virus to that seen with two different passaged virus innocula. In each case, naïve Mane-A*10 negative pigtail macaques were infected with plasma and PBMC derived from further *in vivo* passages of SHIV_mn229_ containing 3–30 fold lower proportions of WT KP9 virus [5]. We also compared the rates of reversion at another Gag epitope, AF9, for which we have also developed a sensitive qRT-PCR for [31].

Outgrowth of WT virus following inoculation with the EM SHIV_mn229_ stock (containing 89% EM virus and 11% WT virus) is almost identical and very rapid in all Mane-A*10 negative animals studied (Figure 1A, Table 1). WT virus grows most rapidly in the early days of infection while the target cells are not yet depleted, to dominate the viral population by the second week of infection. The K165R EM virus decays to very minor or undetectable level by day 56 of infection in all animals.

**Table 1:** Reversion of KP9 and AF9 mutant viruses.

Epitope	Virus	%WT in Inocula	Ratio WT∶EM in Inocula	Recipient Animal Number	Days to First Detect WT	Days until WT>EM	Reversion Rate at 50% WT
AF9	Passaged SHIV_SF162P3_	50%	1∶1	6175	≤6*	6	0.46
				5904	≤6	6	0.22
KP9	SHIV_mn229_	11%	1∶9	4194	≤7	7	0.42
				4301	≤7	14	0.18
				H20	≤7	7	0.35
				1.7105	≤7	14	
KP9	SIV_mac239_	10%	1∶10	3B14	≤6	16	0.20
				9532	≤6	19	0.31
KP9	SHIV_mn229_ passage A	4%	1∶25	6274	11	63	0.32
				6366	11	75	0.35
KP9	SHIV_mn229_ passage B	0.34%	1∶293	6238	8	>75†	0.083**
				6255	8	>75	0.19**
KP9	SIV_mac239_	0%	NA	5504	10	63	0.11
				6176	10	63	0.067
				6273	14	>77	0.064**

*: ≤indicates WT virus detected at first time point sampled.

**†:** >indicates WT virus levels did not exceed EM virus levels by last time point sampled.

**: Reversion rates at 50% for these animals were determined by extrapolation.

To investigate whether the rapid outgrowth of WT virus in the setting of substantial levels of WT virus in the inoculum could be generalized to other Gag epitopes, we studied rates of reversion at the AF9 epitope. We inoculated 2 naïve Mane-A*17 negative pigtail macaques with a passaged virus containing ∼50% WT and 50% EM virus at AF9 [31]. Very similar rapid outgrowth of WT virus was observed using separate specific qRT-PCRs for WT virus or the 6-bp deletion AF9 mutation (Figure 1B). Again, WT virus grows at a rapid rate over the first week and is the dominant species over EM virus within 2 weeks of inoculation. Thus, where both WT and EM viruses are present in substantial quantities in the virus inoculum, the WT virus very rapidly outgrows the EM virus upon transfer to MHC-mismatched hosts.

### Outgrowth of WT virus delayed and slowed with lower levels of WT in inoculum

The rapid outgrowth of WT virus in the previous experiments suggested sufficient quantities of WT virus were present in the inoculum to co-infect the host and then rapidly out-compete the EM virus. We therefore sought to elucidate the impact of much smaller amounts of WT KP9 on reversion at KP9. We first chose to transfer plasma and cells from Mane-A*10+ animals infected for lengthy periods of time with SHIV_mn229_ (essentially further *in vivo* passages of the original SHIV_HXB2_ stock). For these virus transfer experiments, we selected donor Mane-A*10+ animals that were previously vaccinated with SIV Gag-expressing DNA and recombinant Fowlpoxvirus vaccines [9],[28],[29]. The donor animals generated KP9-specific CD8 T cell responses after vaccination, which are further boosted after virus challenge. The KP9-specific responses in the donor animals select the EM virus and further reduce levels of WT virus. By qRT-PCR, the passaged viruses chosen had either only 4% WT virus or 0.34% WT virus at KP9 using our qRT-PCR (Figure 1C and 1D, and Table 1).

The viral transfer was successful, resulting in an infection of all animals studied. The appearance of WT virus was delayed in recipients of both *in vivo* passaged SHIV_mn229_, which contained lower levels of WT virus (Figure 1C and 1D). Although high levels of EM virus were detected within 6 days of transfer with the KP9 qRT-PCR, very low levels of WT virus were detected only by day 8–11 after transfer. Further, even after the detection of WT virus, this variant did not expand dramatically to high levels as seen with the original SHIV_mn229_ stock or the passaged AF9 mutant virus. The WT virus took 63–75 days to exceed EM virus in the case of the 4% WT stock, and WT virus levels never exceeded EM virus levels to 75 days of follow up in the case of the 0.34% WT stock.

The kinetics of reversion to WT were compared by estimating the time taken for 50% WT virus to be reached, using linear interpolation of the log-transformed EM and WT viral loads. In the animals infected with 4% WT virus, it took 63–75 days before WT virus reached 50% of the total virus levels. In the case of the animals infected with the 0.34% WT stock, WT virus levels never exceeded EM virus levels to 75 days of follow up. Thus, lower initial WT level was associated with an increased delay in WT virus outgrowth.

### Infection with escape mutant SIV_mac239_

Our analyses of biologic isolates of X4-tropic SHIV_mn229_ (for KP9) and R5-tropic SHIV_SF162P3_ (for the AF9 epitope) strongly suggested that the levels of WT virus in the inoculum have a major bearing on the time needed for outgrowth of the WT virus. However, it is difficult to completely exclude that these uncloned viral stocks contain quasi-species with mutations at other sites or compensatory mutations (although no clear pattern of such mutations were seen during intensive cloning and sequencing). To avoid these potential confounders we constructed a molecular clone of the K165R KP9 EM virus within SIV_mac239_. This enabled us to evaluate rates of reversion using a separate R5-tropic virus in a very tightly controlled manner. We chose to infect naïve Mane-A*10 negative pigtail macaques using plasmid DNA, an approach we and others have previously used successfully, both with attenuated and WT viruses [32], [35]–[37]. Using clonal proviral DNA to initiate the infection eliminates any possibility of generating alternate viral quasispecies *in vitro* prior to *in vivo* inoculation.

To first determine if infection with a mix of WT and EM SIV_mac239_ conforms to the same general principles observed with SHIV_mn229_ and SHIV_SF162P3_ we inoculated 2 naïve Mane-A*10 negative pigtail macaques using a 90∶10 mix of EM∶WT plasmid DNA. Using our qRT-PCR assay to detect WT or EM virus, both WT and EM virus grew readily for the first 2 weeks but WT virus subsequently rapidly outgrew the EM virus (Figure 1E). The EM virus slowly decayed later in infection. This pattern of outgrowth of WT virus using a 90∶10 EM∶WT mix of SIV was almost identical to that observed with the original 89∶11 EM∶WT SHIV_mn229_ stock (Table 1).

We next evaluated the generation and outgrowth of revertant WT virus following inoculation with pure clonal K165R EM SIV_mac239_ (Figure 1F). Again we inoculated proviral DNA and an infection was readily initiated in all 3 Mane-A*10 negative animals studied. We observed an 8–10 day delay in the appearance of the WT virus which took ≥63 days to exceed levels of the EM virus. The patterns of growth of WT virus and decay of EM virus following infection with 0% WT SIV_mac239_ were strikingly similar to those observed with the passaged SHIV_mn229_ inocula with ≤4% WT virus. A comparison of levels of WT and EM viruses across the 6 strains used is shown in Figure 1G, with the viruses grouped according to whether they have ≥ or <10% WT in the inoculum. The grey shading in Figure 1G highlights the consistent 8–10 day delay in appearance of the WT virus in the low WT virus inocula and rapid outgrowth of WT virus in the high WT virus inocula.

### Modelling reversion of WT virus

**Figure 2:**
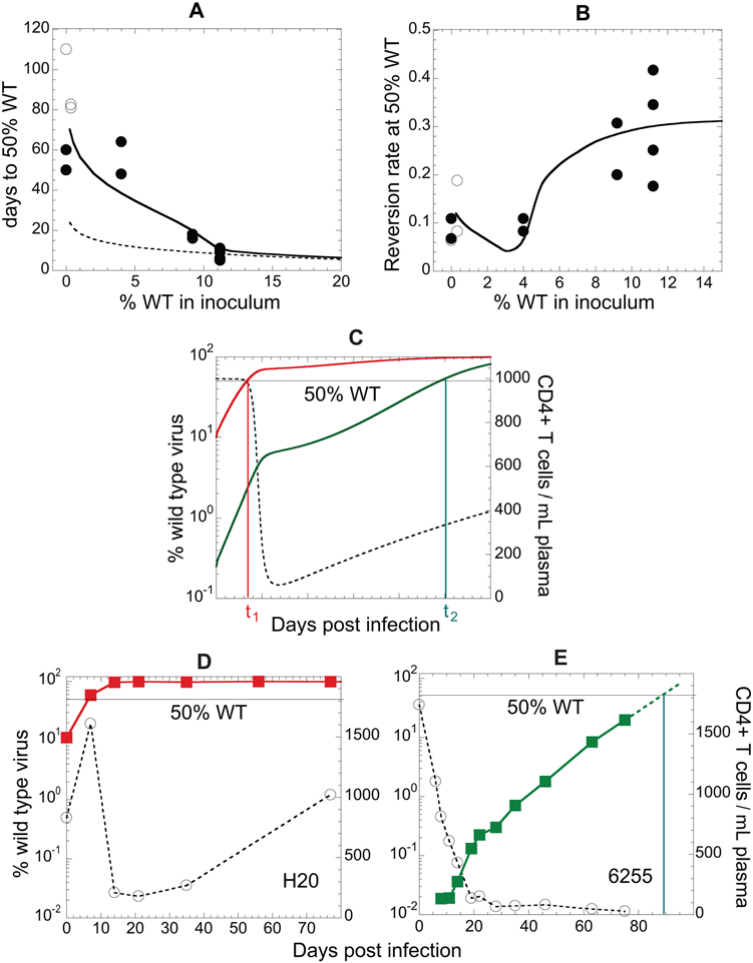
Dependence of reversion dynamics on the percentage of WT in the inoculum. (A) The time needed to reach 50% WT in total viral load depends on the fraction of WT in the inoculum, and starts to increase rapidly with the decrease in initial percentage below approximately 10%. (B) Dependence of reversion rate at 50% WT on the fraction of WT in the inoculum. In (A) and (B): full circle symbols, experimental data; open circles, obtained by extrapolation; line, results of the model for *r_W_*−*r_M_* = 3×10^−4^ µL/cell/day. (C) The observed reversion rate is proportional to the average target cell number (Equation 3). The dashed line represents target cells in time. The red and the green full lines show how % WT in total viral load grows if it is initially 10% or 0.25%, respectively. If WT does not reach 50% before target cells are depleted, then it will take much longer to overtake EM. (D) Experimentally observed CD4+ T cell levels and % WT in the SHIV_mn229_-infected animal H20 (with initial 11% WT at KP9) and (E) in animal 6255, infected with the passaged SHIV_mn229_ with 0.34% WT at KP9 conform to the theoretical pattern in (C).

The experimental analysis above demonstrates that the rate of reversion from WT to EM virus is linked to levels of WT virus in the infecting inoculum. This is not unexpected since, all other factors being equal, a halving of the initial proportion of WT virus would be expected to require one additional doubling time before the WT virus reached 50% of the initial inoculum. However, as illustrated in Figure 2A, the observed effects of reducing the proportion of WT virus are much stronger than expected by this factor alone. In order to understand how the initial WT proportion affects the subsequent dynamics of infection, we modelled the dynamics of WT and EM virus following infection.

From previous work we expect that target cell number (the number of uninfected CD4+ T cells available for infection) is important to viral growth and the rate of reversion [11]. Therefore we expect that early in infection reversion will be extremely rapid, but this will slow towards the peak of infection and in the chronic stage because there are fewer target cells to infect. We analyzed the time taken to reach 50% WT virus using a simple ‘fixed reversion rate’ model (dashed line in Figure 2A). This model illustrates the increase in time to reach 50% WT that we would expect solely from the decrease of WT fraction using a difference in replicative capacities of WT and EM of 3.5×10^−4^ µL/cell/day [5],[10],[31]. We also analyzed a model that takes into account the effects of decreasing viral growth with reduced target cell number (solid line in Figure 2A) using Equation 2, for the same difference in replicative capacities of WT and EM. For an initial WT content between 50 and 10%, the time required for WT to reach 50% of total viral load grows relatively slowly and almost linearly, as expected just from the decrease in WT fraction. However, as the proportion of WT decreases further, this time suddenly increases much more rapidly than expected.

One prediction of this model is that not only will it take longer to reach 50% WT when target cell dynamics are taken into account, but that the reversion rate itself at 50% WT will be slower with lower WT fraction in the inoculum. Indeed, the reversion rate observed at 50% WT is significantly correlated to the fraction of WT in the inoculum (Figure 2B, Table 1, Spearman correlation, p = 0.0005, r = 0.788). The solid line shows the dependence of reversion rate on initial WT fraction predicted by the model Equation 2.

Analysis of the dynamics of WT and EM virus over time explains this effect (Figure 2C). When high initial WT proportion is present, the WT virus outgrows the EM virus in the early phase of infection, when the pool of target cells for the virus is still nearly complete and both viruses are still in an exponential growth phase (solid red line in Figure 2C). Reducing WT virus levels slightly have little effect (just delaying the time to 50% slightly, because of the additional time required for the WT virus to grow). However, once the WT proportion gets below a certain level, it will not reach the 50% level before the peak of infection, and before the extensive depletion of CD4+ T cells (solid green line in Figure 2C). The depletion of target cells slows the growth of both viruses, but importantly also slows the rate at which WT virus overtakes EM virus. If WT virus has not already reached 50% by the time of peak viral load, its rate of progress towards the 50% level is slowed dramatically, and it takes much longer to outgrow EM. The same pattern is observed experimentally, as shown by the examples of animals with initial 11.2% WT (Figure 2D) or with 0.34% WT (Figure 2E).

### Biological implications of slow reversion to WT

**Figure 3:**
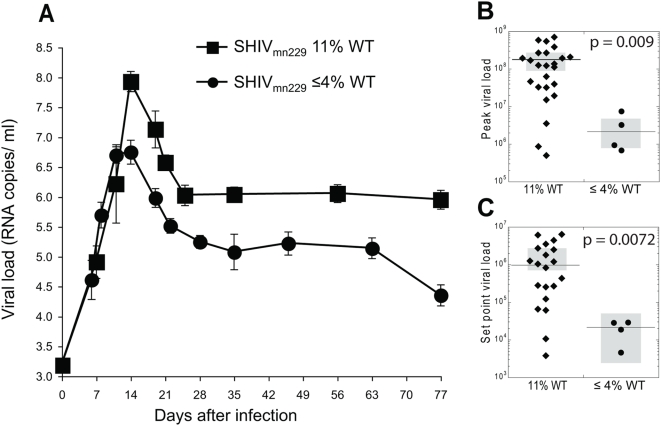
Reduced pathogenicity of passaged SHIV_mn229_. (A) Comparison of mean (±SEM) viral load of 21 animals infected with the same original SHIV_mn229_ stock (89% EM, 11% WT) to four animals inoculated with passaged SHIV_mn229_ isolates containing less WT virus (4% or 0.3%). (B) Comparison of individual peak viral loads. (C) Comparison of individual set point viral loads.

Slow reversion to WT virus implies that the infecting virus population is dominated by the less fit EM virus for longer periods of time in comparison to animals with fast reversion. In theory, this could result in lower viral loads and less pathogenic infections. Goepfert and colleagues recently showed HIV-1 strains transmitted with multiple Gag CD8 T cell escape mutations resulted in overall lower viral loads [7]. However, the direct comparison to otherwise similar virus strains is difficult in humans. Since we had a large series of unvaccinated animals infected with the original SHIV_mn229_ (which reverts rapidly) from previous infection studies [28]–[30] and now multiple animals infected with a further passage of SHIV_mn229_ with less WT virus at KP9 (that reverts slowly), we compared the virologic outcome of infection with both viruses. Both viruses grew effectively and exponentially during acute infection (Figure 3A). However, animals infected with the passaged viruses had much lower content of WT at peak and set point viral loads (average 90% WT at peak in SHIV_mn229_ stock, versus only 5% WT at peak in passaged SHIV_mn229_, and 100% vs. 48% on average respectively at the set point). The increased proportion of WT virus at peak viral load in animals infected with SHIV_mn229_ stock is associated with an increased peak viral load in the animals (Figure 3B): median peak viral load for SHIV_mn229_ stock-infected animals was 1.24×10^8^ (95%CI from 9.6×10^7^ to 2.6×10^8^), and for passaged SHIV_mn229_ it was 2.08×10^6^ (95%CI from 1.9×10^6^ to 8.0×10^6^, Mann-Whitney p = 0.009). Set point viral load was also significantly higher in animals infected with stock virus (Figure 3C): for SHIV_mn229_ stock it was 9.4×10^5^ (95%CI from 7.3×10^5^ to 2.6×10^6^), and for the passaged virus it was 2.3×10^4^ (95%CI from 2.0×10^3^ to 3.8×10^4^, with Mann-Whitney p = 0.0072). Thus, the slow reversion of WT virus in animals infected with a low proportion of WT virus has direct implications for the virological outcome of infection.

## Discussion

We conducted a large series of infections with combinations of WT and CD8 T cell EM SHIV and SIV viruses. Using a sensitive real-time PCR assay to simultaneously quantify levels of EM and WT viremia, we found levels of WT virus in transmitted EM viruses had a marked influence on the timing and rate of reversion to WT. Where substantial amount of WT virus are present in the inoculum, “reversion” (outgrowth of WT virus) is rapid, but if WT virus is minimal or absent in the inoculum, there is a delay in reversion and when reversion begins, it takes much longer to reach the same levels as the EM virus. The consistency of these findings across multiple SHIV and SIV isolates suggest this is a general phenomenon of CD8 T cell EM reversion. This has important consequences for HIV pathogenesis, since we found a delayed and slow pattern of reversion of biologic SHIV quasispecies (where the less fit EM virus dominates the infection for longer periods) is associated with significantly reduced viral loads through to chronic infection. Since virtually every HIV infection involves the selection of EM viruses, transmission of EM viruses or mixes of EM and WT virus will be the norm. Our studies suggest that in the subset of around one quarter of new HIV infections, where 2 or more viruses are transmitted [25], infections will be more pathogenic if WT virus is present at significant levels in the transmitted inoculum. This data provides rigorous experimental support to recent large observational studies in humans, where HIV-1 Gag CD8 T cell escape mutations in the donor were associated with reduced viral loads in recipients [7],[8].

A potential limitation to our findings of reduced viral load following infection with EM SHIV quasispecies with very low levels of WT virus is that we cannot exclude that mutations other that the KP9 K165R EM present, including distant compensatory mutations, in the passaged virus inocula could also be contributing to the reduced VL upon transmission. Indeed, human HIV-1 transmission studies by Goepfert and colleagues suggest that multiple Gag CD8 T cell epitope mutations may result in reduced VLs in the recipients [7]. Since multiple HIV-specific CD8 T cell responses restricted by different HLA class I alleles are typically generated by infected subjects, transmission of viruses with multiple CD8 T cell escape mutations should be the rule, rather than the exception. Our experience with SHIV_mn229_-infected pigtail macaques suggests that the KP9-specific CD8 T cell response is highly immunodominant and the rapidly pathogenic nature of the virus rarely permits generation of detectable responses to other epitopes, suggesting the much lower proportions of WT virus in the passaged SHIV_mn229_ viruses were primarily responsible for the lower viral loads. None-the-less, larger studies of cloned SIV viruses would assist in further defining the role of individual mutations or combinations of CD8 T cell escape mutations in reducing viral pathogenicity.

The obvious question arises as to why outgrowth of WT virus is so slow when it does not proceed during very early acute infection, since the intrinsic fitness cost of the mutation should be the same regardless of the timing of infection. Modelling reveals that reversion rate is proportional to target cell number [11]. This explains why, in majority of cases, we observe the maximum reversion rate early in infection. If WT is initially present in a sufficient amount, so that it reaches 50% of total viral load during the early phase of exponential growth, before target cells are substantially depleted, reversion is rapid. The time needed to overtake EM will increase slowly with decrease in percent of WT in the inoculum. However, for initial WT fractions below a certain threshold, WT will not overtake EM before the peak viral load. In this case, the extensive depletion of target cells will slow down the reversion rate and markedly increase the time to 50% WT.

A caveat to these studies is the study of CXCR4-utilizing SHIV viruses for these analyses. Since these viruses deplete all naïve CXCR4-expressing CD4 T cells, the depletion of peripheral CD4 T cells should approximate total CD4 T cell loss, and thus frequent monitoring of peripheral CD4 T cells allows us to model CD4 T cell depletion and reversion. Typical CCR5-utilizing HIV-1 and SIV strains target memory CD4 T cells which are most abundant in the gastrointestinal tract and rapidly depleted during acute infection. These cells are less amenable to the frequent monitoring required to correlate of the levels of CD4 T cell depletion with the rate of reversion of viral escape mutants. However, previous analysis comparing the dynamics of CD4+ T cell depletion in the blood during SHIV infection and in the gut following SIV infection suggest that the dynamics are remarkably similar [38],[39]. Further analyses of infection of macaques with CCR5-utilizing SIV strains containing CD8 T cell escape mutations and frequent gut biopsies to assess memory CD4 T cell depletion are warranted. Additionally, although CD4 T cells are typically responsible for the majority of virus replication [40], the use of CCR5-tropic viruses would permit a better assessment of the contribution of non-CD4 T cell targets such as macrophages to virus growth and reversion of escape mutant viruses.

Although we studied a modest number of animals within each group, our findings were consistent across both SHIV and SIV infection models using both biological isolates (for the SHIV studies) and molecular clones (for the SIV studies). When WT virus comprised ≥10% of the inoculum, expansion of WT virus during acute infection was exponential and uniform across SHIV_mn229_, SHIV_SF162P3_ and SIV_mac239_ infection models. When WT virus was <4% of the inoculum, there was a consistent delay of 8–11 days until WT virus is first detected even at very low levels. This occurs during the critical window of early virus dissemination, the so-called “eclipse” phase of very early acute HIV-1 infection of humans [25],[41]. Our studies support previous HIV-1 transmission analyses that suggest that when EM variants are primarily transmitted (likely the majority of cases) a less fit EM virus will predominate during acute infection that may be less pathogenic [7],[8]. Our studies suggest “founder” effects of transmission of EM viruses into new hosts could have a bearing on overall disease pathogenesis of HIV-1 and potentially other variable RNA viruses such as Hepatitis C Virus. We speculate that if virus evolution and the emergence of WT virus is further delayed by even partially successful prevention strategies such as vaccination, the infection may result in lower viral loads, delayed disease and reduced forward transmission.
